# Reply to Külls et al., “Residual confounding and outcome definition in observational comparisons of PDR-*Acinetobacter baumannii* therapies”

**DOI:** 10.1128/aac.00104-26

**Published:** 2026-03-16

**Authors:** Konstantinos Pontikis, Ilias Karaiskos, Helen Giamarellou, George L. Daikos

**Affiliations:** 1 Intensive Care Unit 1st Department of Pulmonology, Chest Diseases Hospital "I Sotiria," National and Kapodistrian University of Athens 68993, Athens, Greece; 2 1st Department of Internal Medicine–Infectious Diseases, Hygeia General Hospital, Athens, Greece; 3 2nd Department of Internal Medicine, Mitera General Hospital, Athens, Greece; Houston Methodist Hospital and Weill Cornell Medical College, Houston, Texas, USA

## REPLY

We would like to thank Külls and colleagues for their commentary on our work (1). The authors have summarized the limitations of the DESPAIR study on pan-drug-resistant *Acinetobacter baumannii* infections. While we agree that definitive conclusions cannot be drawn from an observational study with a limited number of patients, a few points merit emphasis.

First, colistin-resistant *Acinetobacter baumannii* infections are currently very common in countries affected by antimicrobial resistance, and there is a complete paucity of data to inform clinical decisions, apart from small case series. Second, it is highly unlikely that a randomized study will be conducted, meaning that the evidence will most likely stem from observational studies, which are collectively prone to bias. To circumvent some of these biases, patient matching is almost imperative, which is what we attempted to do. Inverse probability of treatment weighting (IPTW) is a frequently used method for this purpose and has been employed in seminal studies in the field of antimicrobial-resistant infections (2). At this point, it is worth noting that IPTW propensity matching was effective in adjusting for baseline imbalances in our study population. As Külls et al. note in their commentary, there were significant differences in ICU admission, invasive mechanical ventilation, and ventilator-associated pneumonia between the two groups before matching; however, these differences were accounted for by propensity matching. It should be noted that our matching approach did not rely only on SOFA score, as Külls et al. note, but also additionally on patient disposition and infection type. As a result, in the matched populations (Table S9 in our original article), ward admission was 12.9% and 14.6% (*P*: 0.839, standardized mean difference [SMD]: 0.050); invasive mechanical ventilation use at infection onset was 75.5% and 57.1% (*P*: 0.170, SMD: 0.398); and VAP percentage was 79.5% and 76.9% (*P*: 0.925, SMD: 0.108) in the two groups, respectively. Thus, the adjusted comparisons that further enhanced the treatment effect were performed between two populations in whom most of the baseline imbalances were accounted for.

Third, with regard to the primary outcome, Külls et al. are correct to point out that some constituents are prone to subjective evaluation. However, some of the most influential studies in the field have used almost the same composite outcome (3), which reflects what is pragmatically regarded as failure in clinical practice. Furthermore, secondary analyses of mortality and evolution of multiorgan dysfunction, which are less subjective, point in the same direction (although some comparisons are marginally statistically non-significant), fortifying our main findings.

Fourth, we agree with Külls et al. that extrapolating *in vitro* synergy to clinical practice is an unsafe approach. However, we followed the opposite approach when we tried to explain our findings through basic research. We and others believe that, when present, biologic plausibility strengthens the probability of statistical associations being etiological (4).

Finally, we share the concerns of Külls et al. regarding the adjudication of toxicity in clinical practice. Indeed, our data suggest that renal injury is more common in patients of Group B, while both regimens contained colistin (Table S7 in our original article), a result that merits explanation. We believe that, in some cases, toxicity essentially represents disease progression (e.g., renal injury as a manifestation of multiorgan dysfunction syndrome) rather than true pharmacologic toxicity. In accordance with this, our third sensitivity analysis showed that excluding toxicity withdrawals from our primary endpoint did not alter the results.

To conclude, we believe that Külls et al.’s critique is relevant to observational research as a whole, as it highlights the inherent limitations of retrospective analyses. However, in the field of pan-drug-resistant infections, it is highly likely that studies of this type will continue to provide the best available evidence. In this regard, every effort should be made to handle even the slightest amount of data as efficiently as possible, which we believe we did.
